# Prevalence and Severity of Circumferential Alveolar Bone Loss Using CBCT Images: A Retrospective Study of 20,620 Surfaces of 5155 Teeth

**DOI:** 10.3390/diagnostics14050507

**Published:** 2024-02-27

**Authors:** Sarhang Sarwat Gul

**Affiliations:** 1 Medical Laboratory Department, College of Health and Medical Technology, Sulaimani Polytechnic University, Sulaymaniyah 46001, Iraq; sarhang.hama@uinvsul.edu.iq or sarhang.hama@spu.edu.iq; 2 Department of Periodontics, College of Dentistry, University of Sulaimani, Sulaymaniyah 46001, Iraq

**Keywords:** CBCT image, alveolar bone loss, periodontal disease, prevalence, severity

## Abstract

Periodontal disease is a site-specific disease affecting the supporting tissues of the teeth. It is useful for the clinician to have information about the prevalence and severity of alveolar bone loss (ABL) according to the site, location, and position of the teeth for a better treatment plan and expected prognosis. This study aimed to assess the prevalence and severity of ABL at mesial, distal, buccal and lingual sites of teeth in different locations, positions and sides of the dentition. The ABL of 20,620 sites of 5155 teeth in 212 patients was assessed using cone-beam computed tomography from the cemento-enamel junction to the crest of the alveolar bone. The prevalence of ABL was higher in the interproximal sites as well as anterior and mandibular teeth compared to their counterparts. Buccal sites and anterior teeth revealed higher ABL levels than the other tooth sites and posterior teeth, respectively. Furthermore, associations in the severity of ABL were observed between distal and mesial sites, buccal and lingual sites, maxillary and mandibular teeth, anterior and posterior teeth, and right and left sides. This study showed that the prevalence and severity of ABL differ from one tooth site to another and according to the tooth’s location in the dentition. Higher prevalences were found in the interproximal sites, anterior teeth and mandibular teeth; higher ABL was found in buccal and distal sites, with the strongest associations between distal and mesial sites, buccal and lingual sites, and right and left sides.

## 1. Introduction

The essential characteristic of the destructive periodontal disease is loss of alveolar bone supporting the teeth [1]. Periodontal disease is a chronic and biofilm-mediated inflammatory disease that affects the tooth supporting structure as a result of dysbiosis between the dental biofilm and the host [2]. At the early stage, periodontal disease is called gingivitis and is confined within soft tissues of the periodontium; however, when gingivitis is left untreated with the presence of risk factors, the disease progresses to involve the other periodontal tissues and is called periodontitis [3]. The clinical signs of periodontitis are the presence of inflammation defined by the presence of bleeding on probing, an increased probing pocket depth and attachment loss [1]. In addition, an increased level of alveolar bone loss (ABL) of more than 2 mm is further evidence of periodontitis [4,5].

Nowadays, the prevalence of the severe form of periodontitis is reported to be around 11%; hence, it is considered as the sixth most prevalent disease affecting humankind [6] and one of the leading causes of tooth loss, which significantly affects oral health and quality of life [7,8]. The impact of periodontitis is not limited to the oral health status, as numerous studies reported the association between severe periodontitis and other systemic diseases such as diabetes, cardiovascular diseases and adverse pregnancy outcomes [9,10,11].

Periodontitis is a disease characterized by its site-specific nature, which varies from one tooth to another and one site to another in the same tooth [12]. Diagnosis of periodontal disease relies upon assessment of clinical parameters of bleeding on probing, pocket depth and clinical attachment loss. Moreover, the prognosis of periodontal disease varies based on the presence and extent of the abovementioned clinical parameters on a site-specific basis [13]. It has been reported that the response to periodontal treatment is considerably affected by the depth of the periodontal pocket and the level of clinical attachment loss [14]. In addition, the position of the tooth and location of the disease around the tooth are other factors that influence the prevalence and severity of periodontitis and, consequently, the prognosis of the disease. For example, the teeth opposite the salivary ducts experience a higher prevalence and severity of periodontitis in comparison to the other teeth due to more significant calculus formation, which is considered to be one of the major local risk factors of periodontitis [15]. On the other hand, teeth positioned buccally or lingually usually have a thin alveolar bone plate and are thus more prone to ABL than those correctly positioned within the jaw [16].

Radiography is another tool that is frequently used as an aid to determine the diagnosis of periodontal disease. Indeed, in the latest classification of periodontal diseases and conditions, the severity of ABL is one of the main helpful parameters for diagnosis of periodontal disease and differentiating mild, moderate, and severe forms of periodontitis more correctly [3]. Assessment of ABL is essential to draw a proper plan of periodontal therapy. Thus, measuring the ABL using radiography is a must before treatment planning. Intra-oral periapical radiography and panoramic radiography are the most commonly used types of radiography for determining ABL in dental practice. However, these two radiography techniques have drawbacks such as only providing information on the mesial and distal sites of the teeth due to their two dimensional imaging of the three-dimensional structure of the periodontal tissue, which prevents assessment of buccal and lingual ABL [17]. Moreover, it has been reported that periapical radiography is usually associated with underestimation of ABL of approximately 1.41 ± 2.58 mm [17], whereas panoramic radiography leads to magnification and does not provide accurate detail of the structure, especially in the anterior region, due to overlapping of teeth and other anatomical structures [18]. It is important to acknowledge that minute changes in correctly identifying the cemento-enamel junction and alveolar bone crest will significantly influence the prevalence and severity of periodontal diseases in a population [19]. To address these inherent limitations of periapical and panoramic radiography, a three-dimensional imaging technique, namely cone beam computed tomography (CBCT), has been introduced.

CBCT is regarded as a feasible tool in the field of dentistry to provide high-resolution three-dimensional images with reduced cost, time and radiation in comparison to the conventional CT [20,21]. Further, CBCT has been found to provide highly accurate and consistent data between the marked reference points when compared to the physical examination [21,22]. For example, studies comparing the measurement of ABL in buccal, lingual, mesial and distal sites of teeth by CBCT to intra-surgical intervention revealed no statistically significant differences between them [23,24]. Last but not least, CBCT imaging allows measurement of buccal and lingual ABL by elimination of superimposition of the tooth and anatomical structures [25,26]. Therefore, CBCT can be considered as a valid tool for ABL assessment with 80–100% sensitivity, while the sensitivity of conventional two-dimensional radiographs ranges from 63 to 67% [27].

Examining the site-specific nature of periodontitis using CBCT and correlating the severity of ABL on different sites, locations and positions of teeth in different age groups and gender is of paramount importance for providing clinicians with crucial information necessary during diagnosis of periodontal disease for design of the site-specific treatment plan and expectation of the treatment outcome (prognosis). Although some studies have examined ABL in populations using periapical and panoramic radiographs [28,29], there is a lack of studies using CBCT with large sample sizes to investigate the site-specific nature of ABL in the mesial, distal, buccal and lingual sites, and further investigation is warranted. Therefore, this study aimed to evaluate the prevalence and severity of ABL in all surfaces around the tooth in different age groups and genders. Additionally, the study compares the severity of ABL in different sides (right and left), jaws (maxillary and mandibular) and locations (anterior and posterior).

## 2. Materials and Methods

### 2.1. Study Population

The study protocol was approved by the ethical committee of the College of Dentistry, University of Sulaimani (approval no. 220/23), and in accordance with local data protection guidelines and World Medical Association Declaration of Helsinki of 1975 as revised in 2000. CBCT images for the period between January 2017 and October 2020 were all retrieved from a radiographic archive of a private dental center in Sulaymaniyah, Iraq, and then the extracted data were anonymized. Patients had these CBCTs taken for a variety of reasons, including implant planning, assessing maxillofacial pathology, oral surgical and endodontic reasons. No CBCTs were taken solely for the purpose of this study.

### 2.2. Inclusion and Exclusion Criteria

**Figure 1:**
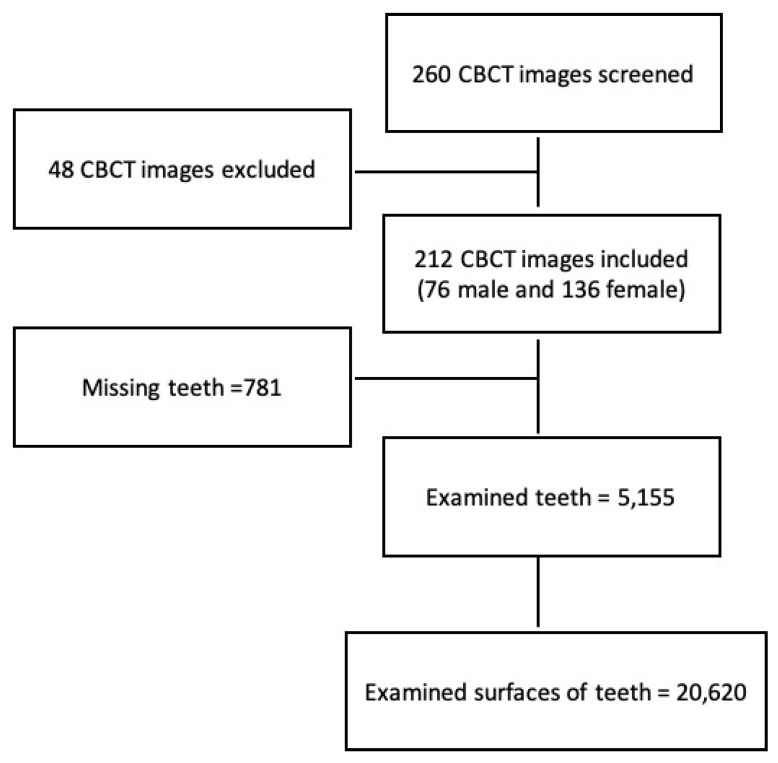
Flow diagram for the CBCT images included in the present study.

Inclusion criteria included undistorted clear CBCT images of full arches for both maxillary and mandibular jaws. Unclear blurred images interfering with the CEJ or that were distorted or overlapped or had any abnormalities that limited the visualization of teeth and bone were excluded as this might have affected the reading of CEJ to ABL. Moreover, images of 3rd molars and those captured for local sections and for patients less than 18 years old were also excluded from the current study [30] (Figure 1).

### 2.3. Acquisition and Measurements of CBCT Images

**Figure 2:**
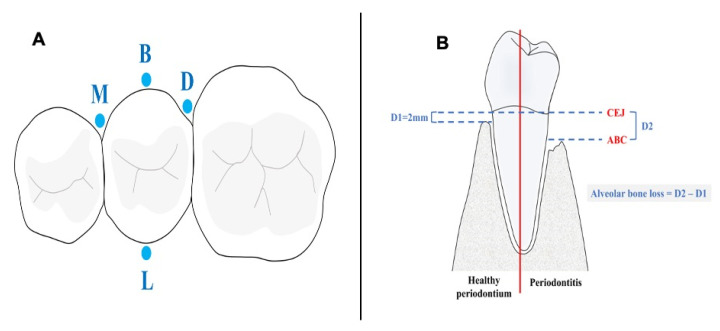
(**A**) The four sites: mesial (M), buccal (B), lingual (L), and distal (D), where the alveolar bone loss was measured. (**B**) Measurement of alveolar bone loss in patients with periodontitis. D1: Distance from CEJ to alveolar bone crest in healthy periodontium. D2: Distance from CEJ to alveolar bone crest in periodontitis subjects. ABL in periodontitis subjects determined by subtracting D1 from D2. CEJ: cementoenamel junction. ABC: Alveolar bone crest.

In this retrospective, cross-sectional study, the ABL was determined at four sites, namely mesial, distal, buccal, and lingual surfaces around each tooth, in the CBCT images (Figure 2A). A digital method of determining ABL on CBCT images was employed using constant anatomic landmarks including the cemento-enamel junction (CEJ) and alveolar bone crest as the reference points, following the method proposed by Mol and Balasundaram [20] in which the severity of bone loss was determined by measuring the distance from the CEJ to the crest of the remaining alveolar bone minus 2 mm at surfaces with a reduced normal level of alveolar bone, as the normal level of ABL is 2 mm apical to CEJ [4,5] (Figure 2B).

**Figure 3:**
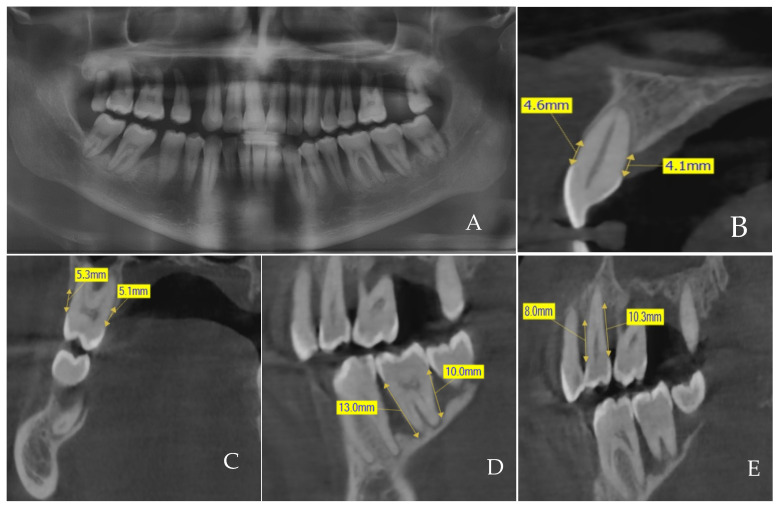
Cone-beam computed tomography images for patients included in the study. (**A**) An overview of the alveolar bone loss by orthopantomography; (**B**,**C**) cross-sectional view for measuring the amount of alveolar bone loss on the buccal and lingual sites; (**D**,**E**) tangential view for measuring the amount of alveolar bone loss on the mesial and distal sites.

The CBCT images acquisition was carried out using a Sirona GALILEOS comfort-2016 set at 98 Kv, 25 mAs, Field of View GALILEOS Compact (12 × 15 × 15) cm^3^ with a 3D resolution (isotropic voxel size) of 0.3 mm according to the manufacturer’s instruction to allow replication and comparison to other studies, such as the study by Zardawi [30]. Measurement of ABL in interproximal sites (mesial and distal sites) was determined at a tangential view, whereas the buccal and lingual levels of ABL were found at cross-sectional views (Figure 3). Descriptive variables including age, sex, number of extracted teeth, and number of surfaces examined were also recorded. Later, the CBCT images were divided into the following seven groups according to the age of the patients: G1 (18–20), G2 (21–30), G3 (31–40), G4 (41–50), while G5 (51–60), G6 (61–70), and G7 > 70 years.

### 2.4. Sample Size Calculation

The sample size for the current study was calculated according to the following formula:

(N = (Zα)^2^ × (p × q)/d^2^)

where Zα is 1.645 with a 90% confidence interval, which is the standard normal probability distribution that puts an area of 0.90 in the center, an area of 0.05 in the far-left tail, and an area of 0.05 in the far-right tail. The prevalence (p) of moderate to severe forms of periodontitis, according to the American Academy of Periodontology/Centers for Disease Control and Prevention, ranges from 24 to 25% [31]. The relative precision (d) was considered as 20% of p to generate a higher sample size, which would ultimately ensure the study data’s accuracy. The calculated sample size was 202 subjects, which was rounded to 212 subjects. 

### 2.5. Inter and Intra Examiner Calibration

A qualified periodontist was trained by an oral and maxillofacial radiologist (PhD in maxillofacial radiology) to read CBCT images and identify the parameters investigated, namely the prevalence of ABL and the degree of ABL in relation to the CEJ and alveolar bone crest. A sample of 10 CBCT scans that were not included in the study were used for training the reader. The results were then discussed with the radiologist, and further training was undertaken 2 weeks later to ensure consistency. Following this training, using an intraclass correlation coefficient test, the inter-examiner calibration and intra-examiner calibration (after 2 week) values were 0.902 and 0.915, respectively. Therefore, the measurement error was found to be insignificant.

### 2.6. Statistical Analysis

The Kolmogorov–Smirnov test was used to test the normality of the data to ensure that appropriate tests (parametric and non-parametric tests) were selected. The prevalence of ABL in all examined sites per tooth was reported. The severity of ABL (mm) was expressed as median and interquartile range (IQR). As the data were non-parametric, the Mann–Whitney test was used to compare the severity of ABL (mm) between males and females, and the two groups were compared. The Kruskal–Wallis test for multigroup comparisons (Dunn’s test) was used to compare the severity of ABL (mm) between the examined sites, the right and left sides, maxillary and mandibular teeth, and between the anterior and posterior teeth. Furthermore, the Bonferroni test was used for multiple comparison correction. Spearman’s correlation test was used to find the association of severity of ABL (mm) between the examined sites and between the right and left sides. A *p* value of ≤0.05 was considered significant for statistical analysis, and data analysis was carried out using the statistical software package IBMSPSS (Statistical Package for the Social Sciences version 22.0, Chicago, IL, USA).

## 3. Results

**Figure 4:**
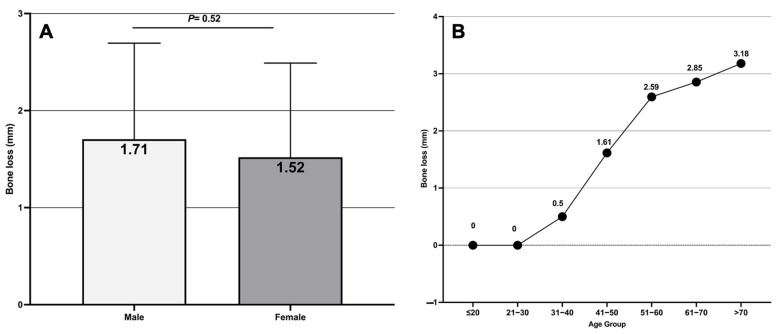
(**A**) Comparison of median alveolar bone loss between males and females showed no statistically significant difference between them; (**B**) assessment of median alveolar bone loss by study age groups indicated that after 30 years of age the alveolar bone loss steadily increases, and the severity of alveolar bone loss is directly correlated with subject age.

A total of 20,620 sites of 5155 teeth from 212 subjects (76 male and 136 female) were examined. The mean age of the study subjects was 40.4 ± 13.3 years old. The number of missing teeth amongst the study population was 781 teeth (mean = 3.68 ± 4.5 teeth). The overall ABL across the study subjects was 1.58 mm (IQR = 0–2.53), with no statistically significant difference between males (1.71, IQR = 0–2.69) and females (median = 1.52, IQR = 0–249) (*p* = 0.52) (Figure 4A). Furthermore, the severity of ABL appeared to increase steadily with the age of the subjects. For example, the median ABL in subjects younger than 30 years old was zero, whereas in subjects aged 31–40 years old and 41–50 years old it was 0.5 mm and 1.61 mm, respectively. The highest amount of ABL was detected in subjects older than 70 years old (median = 3.18 mm) (Figure 4B).

**Figure 5:**
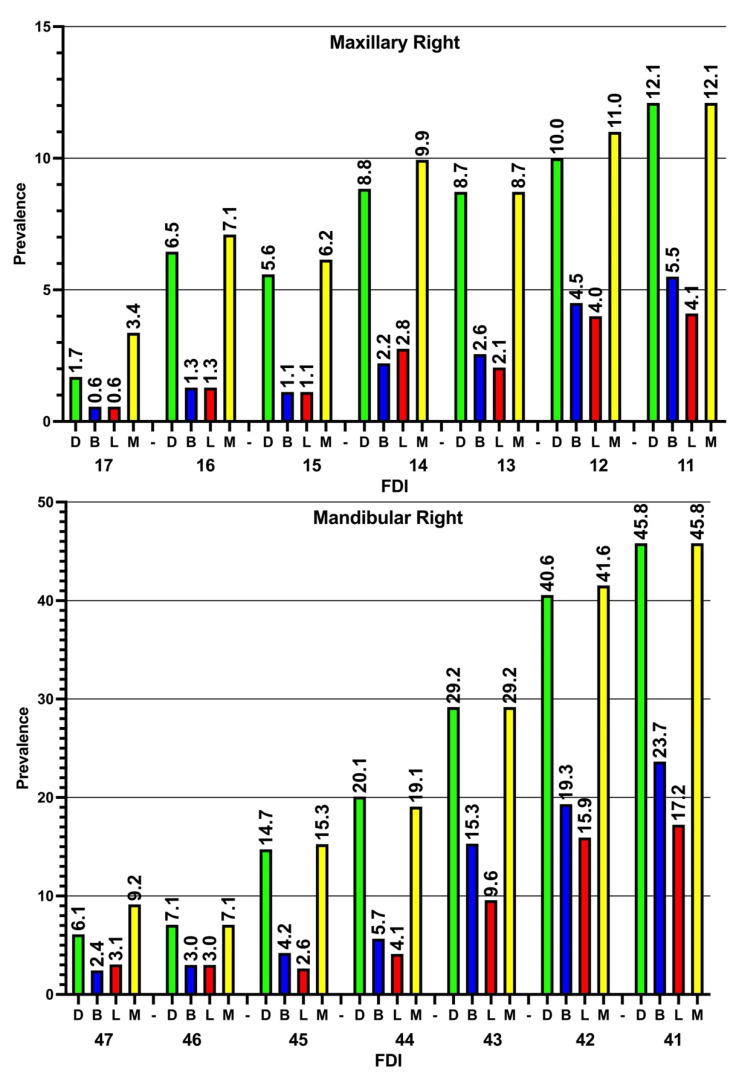
Prevalence of alveolar bone loss in the four examined sites on the right sides of the maxillary and mandibular teeth. M: mesial, B: buccal, L: lingual, D: distal, FDI: FDI tooth numbering system. The prevalence of ABL in all teeth is higher in the mesial and distal sites in comparison to the buccal and lingual sites. Additionally, it is noticeable that the prevalence of ABL is higher in the anterior teeth than the posterior teeth.

**Figure 6:**
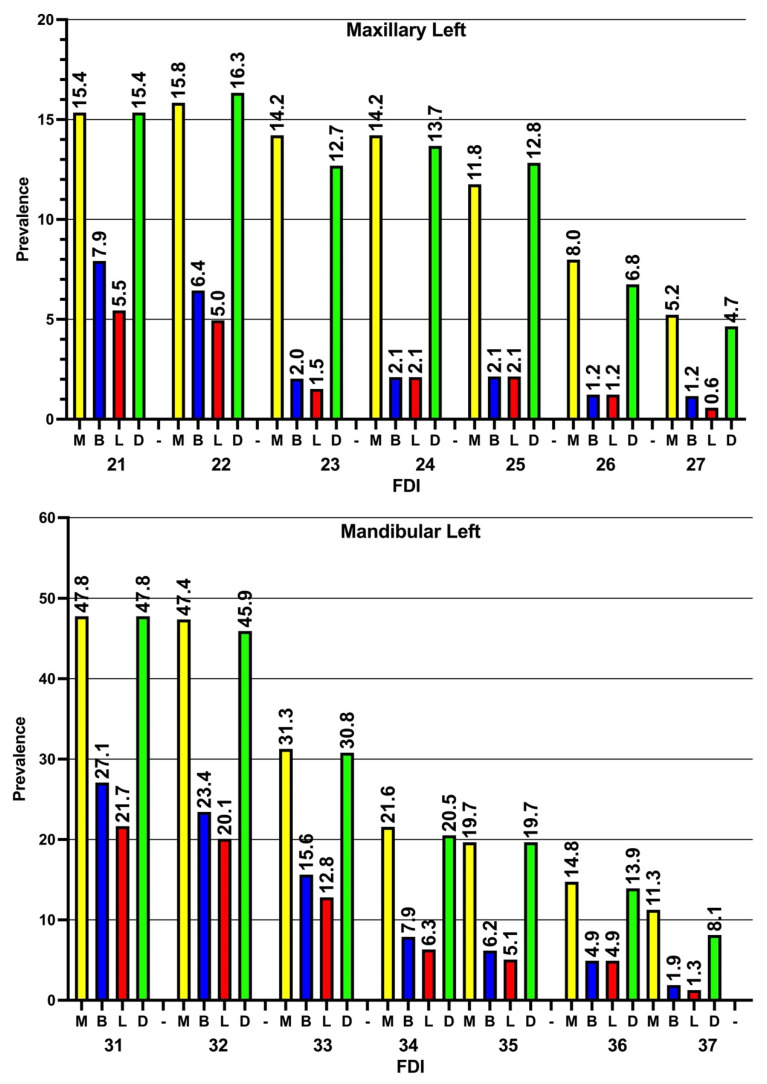
Prevalence of alveolar bone loss (median and IQR) in the four examined sites on the left sides of the maxillary and mandibular teeth. M: mesial, B: buccal, L: lingual, D: distal, FDI: FDI tooth numbering system. Similar to the right side, the prevalence of ABL in all teeth is higher in the mesial and distal sites in comparison to the buccal and lingual sites. Again, it is noticeable that the prevalence of ABL is higher in the anterior teeth than the posterior teeth.

The prevalence of ABL in the four examined sites of teeth was investigated, and the prevalence of ABL was found to be higher in interproximal (mesial and distal) sites than in buccal and lingual sites. In general, it can be noted that the prevalence of ABL tended to increase in both the maxillary and mandibular anterior teeth in comparison to the posterior teeth. Additionally, the prevalence of ABL was found to be higher in the mandibular teeth than the maxillary teeth (Figure 5 and Figure 6).

**Figure 7:**
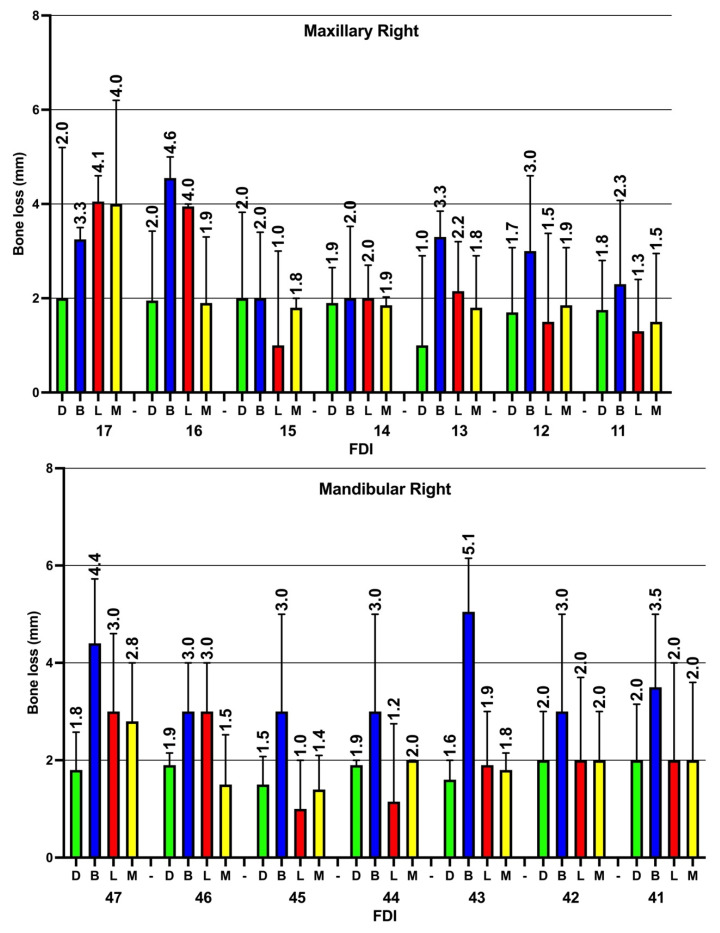
Severity of alveolar bone loss (median and IQR) in the four examined sites on the right sides of the maxillary and mandibular teeth. M: mesial, B: buccal, L: lingual, D: distal, FDI: FDI tooth numbering system. The figure shows that the severity of ABL in all teeth is always higher in the buccal sites compared to other sites; moreover, the severity of ABL in mandibular teeth is usually higher than in their maxillary counterparts.

**Figure 8:**
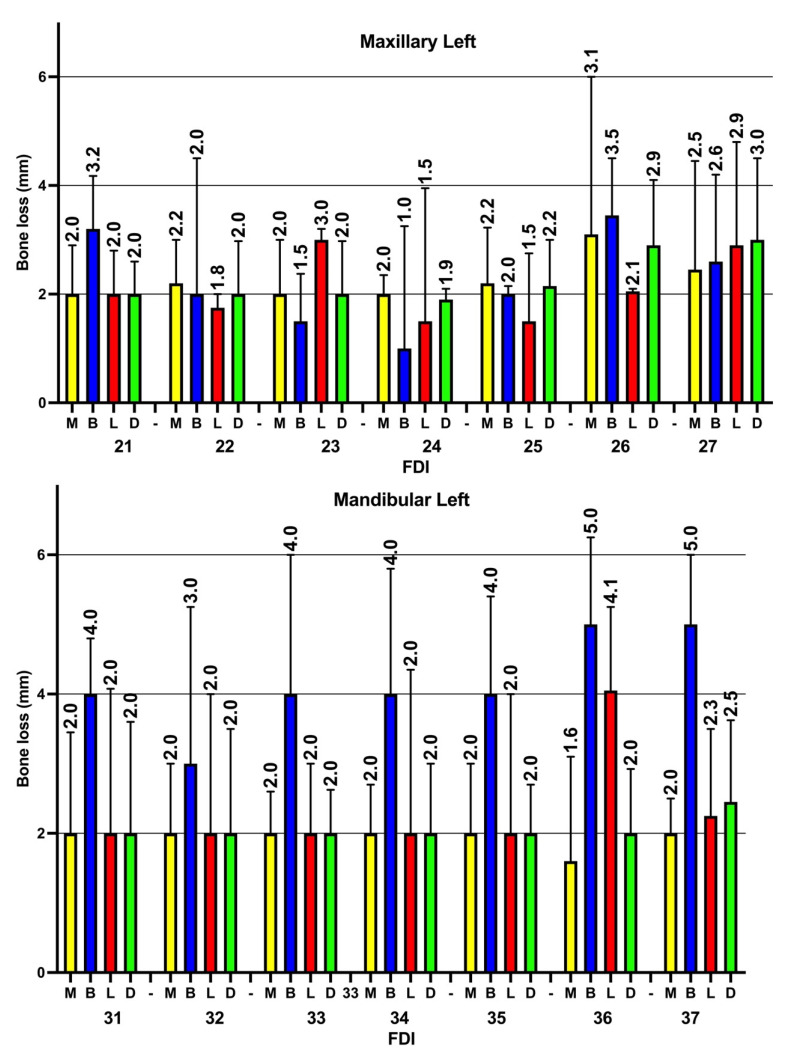
Severity of alveolar bone loss (median and IQR) in the four examined sites on the left sides of the maxillary and mandibular teeth. M: mesial, B: buccal, L: lingual, D: distal, FDI: FDI tooth numbering system. Similar to the right side, the severity of ABL in mandibular teeth (especially at buccal site) is usually higher than in their maxillary counterparts.

In addition, when the current study examined the severity of ABL in the four sites around the teeth, although the prevalence of ABL was found to be higher at both the mesial and distal surfaces, the severity of alveolar bone loss was higher in the buccal and lingual tooth sites in comparison to the mesial and distal sites. Again, the severity of ABL was higher in both the posterior maxillary and mandibular teeth in comparison to the anterior teeth (Figure 7 and Figure 8).

The severity of bone loss at different tooth sites was examined, and the results show statistically significant differences in the severity of ABL when comparing the distal vs. buccal sites (*p* < 0.05); additionally, a statistically significant difference was apparent between the distal and a combination of buccal and lingual sites (*p* < 0.05). However, no statistically significant differences were found when comparing distal vs. mesial surfaces and distal vs. lingual surfaces (*p* > 0.05). On the other hand, buccal sites revealed higher levels of ABL (median = 3.5 mm, IQR = 2–5) when compared to lingual (median = 2, IQR = 2–3.8), mesial (median = 2 mm, IQR = 1–3) and interproximal (median = 2 mm, IQR = 1–3) sites, and these differences were statistically significant (*p* < 0.05). Furthermore, no statistically significant differences were found when lingual sites were compared to mesial and interproximal sites.

In the present study, when the levels of ABL were compared between right and left sides as well as between maxillary and mandibular teeth, the results showed no statistically significant differences between right and left sides (*p* = 0.0889) or between maxillary and mandibular teeth (*p* = 0.1474). However, when comparing levels of ABL between anterior (median = 11 mm, IQR = 0–33) and posterior (median = 4 mm, IQR = 0–9.97) teeth, a statistically significant difference was detected (*p* < 0.0001) (Table 1).

**Figure 9:**
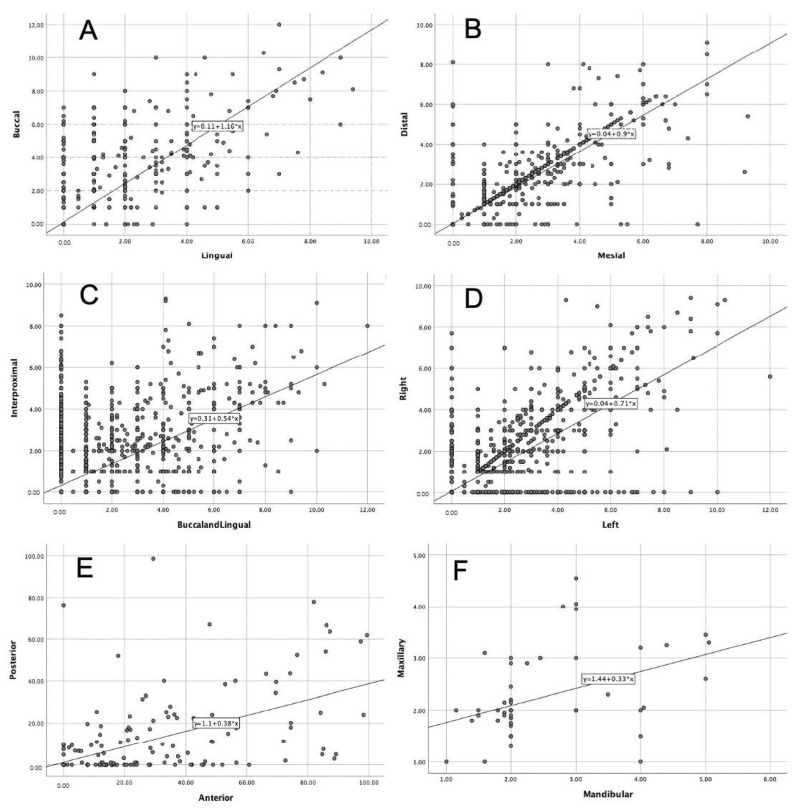
Correlation of the alveolar bone loss between (**A**) buccal and lingual sites, (**B**) distal and mesial sites, (**C**) interproximal with buccal and lingual sites, (**D**) right and left sides, (**E**) anterior and posterior teeth and (**F**) maxillary and mandibular teeth. A statistically significant correlation was identified between all examined sites, sides, locations and position of the teeth. The greatest correlation was found between buccal and lingual sites (r = 0.96, *p* = 0.0001), followed by distal and mesial sites (r = 0.865, *p* = 0.0001). Conversely, the lowest correlations were found when comparing interproximal sites to buccal and lingual sites (r = 0.477, *p* = 0.0001), followed by the correlation between maxillary and mandibular teeth (r = 0.46, *p* = 0.002).

The association of severity of ABL with different sites of the teeth was investigated, and the results show a highly statistically significant correlation between buccal and lingual sites, on the one hand (r = 0.96, *p* = 0.0001), and distal and mesial sites (r = 0.865, *p* = 0.0001), on the other hand (Figure 9A,B, Table 2). In addition, the results show a statistically significant correlation between interproximal surfaces and combination of buccal and lingual sites (r = 0.477, *p* = 0.0001) (Figure 9C). It is worth noting that the levels of association between buccal or lingual (r = 0.96) and interproximal (mesial and distal) sites (r = 0.865) are higher than the association between the interproximal surfaces and combination of buccal and lingual sites (r = 0.477) (Table 2).

As the study aimed to evaluate the overall characteristics of ABL in the study sample, the associations of ABL level with different sides of the mouth (right and left), maxillary and mandibular teeth and anterior and posterior teeth were examined. Similarly, statistically significant associations were found between left and right sides (r = 0.815, *p* = 0.0001) (Figure 9D), followed by anterior and posterior teeth (r = 0.669, *p* = 0.0001) (Figure 9E), and later between maxillary and mandibular teeth (r = 0.46, *p* = 0.002) (Figure 9F, Table 2). Finally, the correlation equations between the abovementioned tooth sites, sides and locations of the teeth (maxillary and mandibular, anterior and posterior) were calculated as presented in Table 2.

## 4. Discussion

The prevention and treatment of periodontal disease rely upon different approaches which involve implementing a preventive program to reduce the prevalence of periodontitis in a population and providing tailored patient treatment plans for patients with already developed periodontitis and addressing the risk factors of the disease [32]. The latter approach is of great value from a cost–benefit point of view as it directs the treatment to the area that requires more attention instead of providing universal and random treatment for all the tooth sites. It is important, therefore, to have information about the rate of progression of ABL for different age groups, sexes, sites per tooth, locations, positions and sides within dentition. Moreover, the association of the level of ABL at the aforementioned sites within the dentition would help the clinician to design a treatment plan better tailored to the patient. However, few studies have examined ABL as an important parameter of periodontal disease, in the required detail, with a sufficient sample size, and using a reliable technique such as CBCT. Accordingly, this retrospective study set out to assess the prevalence and severity of ABL at four tooth sites and across the whole dentition. In addition, the associations of ABL with different areas of the mouth were explored.

In the latest classification of periodontal diseases and conditions, the presence and extent of ABL is one of the key parameters to differentiate between gingivitis and periodontitis, on the one hand, and the severity of periodontitis (mild, moderate and severe), on the other hand [3]. In this study, since the normal position of the alveolar bone crest in healthy periodontal tissue was reported to be 2 mm below the CEJ [4,5], a 2 mm cut off point for ABL was used. Certainly, an inaccurate definition of the cut off point for ABL would highly influence the prevalence and severity of periodontal disease. Additionally, the level of ABL was determined by measuring with three-dimensional CBCT, which has shown to be a more accurate and reliable method when compared to two-dimensional radiography [28,29].

It has been reported that the partial mouth periodontal examination protocol cannot accurately reveal the prevalence of periodontal disease. A partial mouth examination protocol tends to underestimate the true prevalence of the disease [33]. Therefore, in the present study, full mouth data on the ABL were used to determine the prevalence and severity of periodontal disease.

The results of this study showed a gradual increase in the ABL with the age of the patient. The median of ABL in individuals of ≤30 years old was shown to be 0 mm, whereas this level increased from 0.5 mm in the 31–40 age group to 3.18 mm in the >70 years age group. This result is commensurate with previous studies that showed ABL to be associated with aging [30,34]. In this study, no statistically significant differences were observed between the two sexes, which is similar to another study [35]. Other studies reported that males have greater risk of ABL than females [36,37]. This can be attributed to the sample population and the methods used to measure the ABL.

This study showed that the prevalence of ABL was higher at the interproximal sites than the buccal and lingual sites. Similarly, the prevalence of ABL was higher in the anterior and mandibular teeth than the posterior and maxillary teeth, respectively. However, the severity of ABL did not follow the same pattern. Here, it is important to acknowledge that although both prevalence and severity depend on measurement of ABL, the methods used to determine each of them are different. For example, the mere presence of ABL of more than 2 mm is considered to indicate the presence of periodontitis, whereas severity is determined by the extent of ABL beyond this 2 mm. This could explain the discrepancies between the prevalence and severity of ABL at different sites, locations, positions and sides in the present study.

The severity of ABL in the buccal sites was higher than in all other examined tooth sites apart from the distal site, which is in agreement with the result reported in another study [38]. This can be related to the fact that ABL in the buccal site can be highly affected by the tooth position (where teeth are inclined in buccal direction) [16] and more prone to traumatic brushing than other sites [39]. This is in agreement with other studies that showed the highest level of ABL at the distal sites [34]. On the other hand, the levels of ABL at mesial and distal sites were similar. Again, this can be associated with the anatomical structure of the interproximal area, where there is a lack of keratinized epithelium in the gingival-COL area and plaque control is difficult [40].

Contrary to the higher prevalence of ABL in the anterior teeth than the posterior teeth, the severity of ABL in posterior teeth was found to be significantly higher than in the anterior teeth. A higher prevalence of ABL in the anterior teeth than the posterior teeth was also reported in another study [41]. However, as mentioned earlier, there are methodological differences in determining the prevalence and severity of ABL. The presence of additional risk factors such as caries, overhang of margin restorations in the posterior teeth, and difficulties in providing oral proper oral hygiene measures to the posterior teeth may increase the severity of ABL [42]. These factors might also explain the non-statistically significant differences between the maxillary and mandibular teeth, and right and left sides.

Although periodontal disease is a site-specific disease [13] and the results of this study indicate that both the prevalence and severity of ABL vary, significant associations with the severity of ABL emerge when distal vs. mesial sites, buccal vs. lingual sites, anterior vs. posterior, maxillary vs. mandibular and right vs. left sites are compared. These relationships may partly be explained by the fact that the severe form of periodontal disease is usually found in specific groups of patients that are susceptible to the disease and, similarly, some patients would not develop periodontitis even in old age [43]. In this context, the clinician should be aware that when ABL is detected in a specific area, further examinations are required before deciding on the final diagnosis and treatment plan. The association equations developed in this study might help clinicians to obtain a better overview about the extent and severity of ABL when the disease is present in the generalized form. 

The current study has limitations in that it is solely radiographic and retrospective in nature, and testing the validity through comparison to the clinical periodontal parameters was thus not possible. Furthermore, due to unavailability of background data on the participants, detailed analysis of risk factors such as diabetes and smoking in relation to the prevalence and severity of ABL was not performed. Nevertheless, this study examined the full mouth data at four sites per tooth, with a sufficient sample size and a reliable radiographic technique (CBCT), which makes this study unique in nature through providing in-depth information about the prevalence and severity of ABL in a population. Further, associations of ABL with different sites around the tooth, location (maxillary and mandibular jaws), position (anterior and posterior) and sides (right and left sides) were investigated, and equations were developed. This information is of paramount importance in providing clinical practitioners with an overview of the nature of ABL in a population and what clinical examinations need to be performed in the clinic in terms of diagnosis and providing a tailored treatment plan for the patient. 

## 5. Conclusions

This study has identified that the prevalence and severity of ABL vary according to the tooth site and location, position and side of the tooth. The interproximal sites, anterior teeth and mandibular teeth have a higher prevalence of ABL than their counterparts. Meanwhile, the severity of ABL was higher at the buccal sites, followed by distal sites, and in posterior teeth compared to their counterparts. Furthermore, the results of this study suggest a strong association of severity of ABL between distal and mesial sites, buccal and lingual sites, and right and left sides. 

In summary, the results of the current study suggest that the prevalence and severity of ABL vary according to the tooth site, location and position of the tooth and between maxillary and mandibular teeth. The findings of this study contribute in many ways to our understanding of circumferential ABL across the whole dentition. Further studies with a prospective design are recommended to find the association of ABL with clinical periodontal parameters of probing pocket depth and clinical attachment loss. Additionally, future studies should aim to elucidate the role of major risk factors of periodontal disease, such as diabetes and smoking, in the prevalence and severity of ABL. 

## Figures and Tables

**Table 1:** Comparison of alveolar bone loss at different sites, locations, positions, and sides of the teeth.

Tooth Sites and Location	Median (IQR) mm	Tooth Sites and Location Comparison	Mean Rank 1	Mean Rank 2	Mean Rank diff.	*p* Value
Distal	2 (1–3)	Distal vs. Buccal	3661	5347	−1687	<0.0001
Mesial	2 (1–3)	Distal vs. Lingual	3661	3805	−144.2	>0.9999
Buccal	3.5 (2–5)	Distal vs. Mesial	3661	3716	−54.77	>0.9999
Lingual	2 (2–3.9)	Distal vs. Buccal and Lingual	3661	4658	−997.5	<0.0001
Interproximal	2 (1–3)	Buccal vs. Lingual	5347	3805	1542	<0.0001
Buccal and lingual	3 (1.5–4.3)	Buccal vs. Mesial	5347	3716	1632	<0.0001
Right	2 (1–3)	Buccal vs. Interproximal	5347	3688	1659	<0.0001
Left	2 (1.2–3.5)	Lingual vs. Mesial	3805	3716	89.45	>0.9999
Maxillary	2 (1.8–2.8)	Lingual vs. Interproximal	3805	3688	116.9	>0.9999
Mandibular	2 (2–3)	Mesial vs. Buccal/Lingual	3716	4658	−942.7	<0.0001
Posterior	4 (0–9.97)	Interproximal vs. Buccal/Lingual	3688	4658	−970.1	<0.0001
Anterior	11 (0–33)	Right vs. Left	3812	4072	−260.2	0.0889
		Maxillary vs. Mandibular	4262	3856	−324	0.1474
		Anterior vs. Posterior	3154	4919	−1766	<0.0001

*p*: Kruskal–Wallis test.

**Table 2:** Correlation of alveolar bone loss between tooth sites, right and left sides, anterior and posterior teeth, and maxillary and mandibular teeth.

Tooth Sites and Locations	r	*p* Value	Equation
Buccal	0.96	0.0001	Buccal = 0.11 + 1.16 × Lingual
Lingual			
Distal	0.865	0.0001	Distal = 0.04 + 0.9 × Mesial
Mesial			
Interproximal	0.477	0.0001	Interproximal = 0.31 + 0.54 × Buccal and Lingual
Buccal and lingual			
Right	0.815	0.0001	Right = 0.04 + 0.71 × Left
Left			
Posterior	0.669	0.0001	Posterior = 1.1 + 0.38 × Anterior
Anterior			
Maxillary	0.46	0.002	Maxillary = 1.44 + 0.33 × Mandibular
mandibular			

r: Spearman’s correlation.

## Data Availability

The data are available from the corresponding author upon reasonable request.
